# Protective Effects of Topical Application of Nitrite on Testicular Ischemia-Reperfusion Injury in Rats

**DOI:** 10.1155/2021/5514537

**Published:** 2021-06-26

**Authors:** Jae Won Lee, Ee Taek Hwang, Jin Soo Han

**Affiliations:** ^1^Korea Conformity Laboratories, 8, Gaetbeol-ro 145 beon-gil, Yeonsu-gu, Incheon 21999, Republic of Korea; ^2^Department of Food Biotechnology, Dong-A University, 37, Nakdong-daero 550 beon-gil, Busan 49315, Republic of Korea; ^3^The Institute for the 3Rs & Department of Laboratory Animal Medicine, College of Veterinary Medicine, Konkuk University, 120, Neungdong-ro, Gwangjin-gu, Seoul 05029, Republic of Korea

## Abstract

Testicular torsion is a urologic emergency induced by torsion of the spermatic cord, interrupting blood circulation to the testis. Therapeutic options for testicular torsion, except surgical restoration of testis, are rarely applied in clinical practice. This study, therefore, investigated whether topical application of nitrite (NO_2_^−^) is beneficial in tissue damage due to testicular ischemia-reperfusion (I/R) injury in rats. Pubertal Sprague-Dawley rats were assigned to seven groups: group A, sham-operated control group; group B, I/R with no treatment; groups C, D, and E, I/R followed by treatment with three different doses of nitrite; group F, I/R followed by administration of nitrite and a NO scavenger, C-PTIO (2-(4-carboxyphenyl)-4,4,5,5-tetramethylimidazoline-1-oxyl-3-oxide potassium salt); and group G, I/R followed by administration of nitrate (NO_3_^−^). Unilateral testicular ischemia was maintained for 5 h, followed by reperfusion for 24 h. Nitrite and nitrate were topically administered before reperfusion. Compared to group A, germ cell apoptosis, oxidative stress, antioxidant enzymatic function, and lipid peroxidation were significantly increased, along with abnormal morphology and impaired spermatogenesis in group B (*P* < 0.05). In contrast, testicular damage was generally attenuated in the nitrite treatment groups due to a reduction in superoxide and peroxynitrite levels and the inhibition of caspase-3-dependent apoptosis (*P* < 0.05 vs. group B). These therapeutic effects of nitrite-derived NO were suppressed after injection of C-PTIO, which showed in group F. Taken together, our results demonstrate that topical application of nitrite may be one of the therapeutic strategies for testicular ischemia-reperfusion injury.

## 1. Introduction

Testicular torsion is a urologic emergency that is primarily induced by torsion of the spermatic cord in infants and children [1]. The basic pathophysiology of testicular torsion-detorsion is ischemia/reperfusion (I/R) injury of the testis which results in increased formation of reactive oxygen species (ROS), leading to DNA damage, endothelial injury, oxidative stress, germinal cell necrosis, testicular apoptosis, and impaired spermatogenesis [2–4].

Nitric oxide (NO) is a vital regulator with (patho)physiologically multifunctional effects, such as modulating vascular activity, cell metabolism, and homeostasis. NO is formed from L-arginine by nitric oxide synthases, endothelial NOS (eNOS), neuronal NOS (nNOS), and inducible NOS (iNOS) [5]. Nitrite is an endogenous molecule formed by the oxidation of nitric oxide under normoxic conditions and is a major circulating and tissue storage form of NO. [5, 6] Conversely, during I/R injury and acidic states, nitrite reductase activity causes the reduction of NO_2_^−^ to NO by several mechanisms, including acidic disproportionation, xanthine-oxidoreductase (XOR), deoxyhemoglobin, and deoxymyoglobin [5, 7–9].

Data on the *in vivo* effects of nitrite-derived NO are still controversial [10–12]. Some investigations have reported that the application of nitrite has ameliorative effects on heart, liver, and brain I/R injury [5, 7, 13], while NO has deleterious effects due to peroxynitrite production by the complex formed between high levels of NO and superoxide radicals during the reperfusion period, which leads to apoptosis and oxidant stress [14]. Therefore, it is necessary to clarify the relationship of NO generation and cytoprotection and to determine the concentrations, routes, and timing of nitrite and nitrate application to harness their therapeutic benefits.

In this study, we hypothesized that direct topical application of nitrite/nitrate to testicular surface may be a therapeutic strategy in testicular I/R. Although it was reported that intravenous or intraperitoneal administration of nitrite protects rat tissue against I/R injury [6, 13], we conducted this further study to confirm the maximized efficacy of NO via direct topical application to I/R injured tissue. Because nitrite administered via systemic application is quickly reduced into NO before it arrives at the testis, we determined to apply topical administration which is expected to be easily applicable to surgical field by clinicians. To investigate this hypothesis, we evaluated the levels of malondialdehyde (MDA) and cyclic guanosine monophosphate (cGMP), antioxidant enzymatic activity, apoptosis, oxidative stress, and spermatogenesis in a testicular I/R injury model.

## 2. Materials and Methods

### 2.1. Animals and Study Design

Animal care and experimental procedures were approved by the Konkuk University Institutional Animal Care and Use Committee. Male Sprague–Dawley rats (weight 150 to 170 g; 6 weeks old) were obtained from Orient Bio Inc. (Gyeonggido, Korea). Animals were housed in individual, ventilated cages with beta-chip bedding on a 12 h : 12 h light : dark cycle. The room temperature was 23 ± 1°C, and relative humidity 50 ± 5%. Gamma sterilized food (Feed lab, Korea) and autoclaved tap water were provided *ad libitum*.

Pubertal rats were randomly assigned to seven groups (10 rats/group). After following a scrotal midline incision under Zoletil® (40 mg/kg) and xylazine (5 mg/kg) anesthesia, the left testis was exteriorized. In sham-operated control group A, the testis was promptly placed back into the scrotum, and 4-0 silk suture was placed through the tunica albuginea. In the remaining six groups, the left testis was rotated 720° in a clockwise direction and maintained in an ischemic state by fixing the testis to the scrotum with a 4-0 silk suture (Ailee, Busan, Korea). After five hours of ischemia, the testis was released in a counterclockwise direction to initiate reperfusion in group B (I/R with no treatment) [7]. Nitrites were administrated topically to rats in groups C, D, and E (0.12, 1.2, and 12 nmol/g body wt, respectively, 1 min before reperfusion). Nitrite (0.12 nmol/g body wt) and C-PTIO (0.01 *μ*mol/g body wt, 5 minutes before ischemia via intravenous injection) were administrated to rats in group F. Nitrate was also administrated topically to rats in group G (0.12 nmol/g body wt, 1 min before reperfusion). Detailed information for the study scheme is shown in Table 1.

Sodium nitrite and sodium nitrate were obtained from Sigma-Aldrich (St. Louis, MO, USA; catalog number S2252 and S8170, respectively). The NO scavenger C-PTIO (2-(4-carboxyphenyl)-4,4,5,5-tetramethyli midazoline-1-oxyl-3-oxide potassium salt) was purchased from Alexis Biochemicals (San Diego, CA, USA; catalog number ALX-430-001).

After surgery, ketoprofen (5 mg/kg) was injected subcutaneously for reducing pain. All animals experienced 24 h of reperfusion, after which a bilateral orchiectomy was performed for subsequent analysis.

### 2.2. Histopathological Evaluation

#### 2.2.1. Spermatogenesis

At necropsy, testicular tissues were removed from the rats and fixed in Bouin's solution. And the testes were dehydrated with the increased ethanol series. Paraffin-embedded tissue was cut into approximately 4 *μ*m thickness with the coronal cross section in the midline area of the testis. Sections were subjected to hematoxylin and eosin (H&E) to evaluate spermatogenesis (×200 field area).

Both the number of germinal cell layers and Johnsen scores were used to categorize spermatogenesis in the testes by counting 10 consecutive seminiferous tubules and calculating the mean number. Each tubular section was given a score ranging from 10 to 1 according to Johnsen's scoring system [9], which is based on the degeneration of germinal epithelium and the presence of germinal cells in the seminiferous tubules. Mean seminiferous tubule diameter (MSTD) was determined from 20 tubular diameters. A testicular MSTD below 260 *μ*m was considered a pathologically low value [15].

#### 2.2.2. Analysis of Oxidative Stress Markers

Paraffin-embedded 4 *μ*m thick sections were deparaffinized and heated in citrate buffer (0.01 M) in a microwave for 10 min for antigen retrieval. The sections were incubated in 3% H_2_O_2_ in methyl alcohol for 30 min to block endogenous peroxidase activity and washed three times in phosphate-buffered saline (PBS). After incubating with blocking serum for 1 h, tissue sections were treated overnight at 4°C with anti-3-nitrotyrosine (3-NT) antibody (Upstate Biotechnology, Lake Placid, NY, USA), a marker of peroxynitrite generation, and subsequently with the secondary antibody, DyLight 405-conjugated AffiniPure Goat Anti-Rabbit (Jackson ImmunoResearch Laboratories, West Grove, PA, USA).

In the presence of superoxide, dihydroethidium (DHE) is rapidly oxidized to ethidium bromide (EtBr), which binds to DNA and emits red fluorescence [16]. Briefly, frozen, enzymatically intact testes in OCT compound (Leica, Bensheim, Germany) were cut into 7–10 *μ*m thick sections and mounted on coating glass slides (HistoBond; Bad Mergentheim, Germany). The tissues were incubated with 5 mM dihydroethidium (DHE) stabilized in diethyl sulfoxide (Invitrogen, Carlsbad, CA, USA; catalog number D23107) at a 1 : 10,000 dilution in PBS and stained in a dark, humidified chamber for 5 min at 37°C. Fluorescent images were obtained using a confocal laser scanning microscope (LSM 710 confocal microscope, Carl Zeiss MicroImaging) and subsequently analyzed using the LSM 710 ZEN software.

We randomly selected five fields per slide and five slides per animal. 3-NT-and DHE-positive stained cells in randomly chosen field were evaluated under the light microscope at a magnification ×200. It was manually counted and averaged for 20 seminiferous tubules.

### 2.3. Biochemical Evaluation

#### 2.3.1. Apoptosis

Frozen testis tissue was homogenized using an IKA T10 basic, Ultra-Turrax homogenizer (IKA-Werke, Staufen, Germany). Homogenates were centrifuged at 4°C at 10,000 × g for 10 min, and the supernatants were stored at -80°C. Samples were diluted with reducing sample buffer and boiled for 10 min at 95°C. Samples resolved by sodium dodecyl sulfate polyacrylamide gel electrophoresis (SDS-PAGE) gel were transferred onto Whatman Protran Nitrocellulose Membranes (Whatman GmbH, Dassel, Germany). After blocking with 5% blocking solution (nonfat dry milk/TBST (Tris-Buffered Saline and Tween)), the membranes were incubated overnight at 4°C in 2.5% blocking solution containing primary antibodies, *β*-actin (Applied Biosystems, CA, USA), PARP (Thermo scientific, USA), and caspase-3 (Abcam, USA). The membranes were incubated with secondary antibodies diluted in 2.5% blocking solution for 1 h.

After washing with TBST, protein was detected using an enhanced chemiluminescence substrate (ELPIS Biotech, Taejon, South Korea). Protein levels were determined with a densitometer (LAS-3000; Fuji Photo Film, Tokyo, Japan), using Science Laboratory 2001 Image Gauge software (version 3.1; Fuji Photo Film, Tokyo, Japan).

#### 2.3.2. Lipid Peroxidation

For measuring MDA content, which is an end-product of lipid peroxidation, the thiobarbituric acid-reactive substance (TBARS) assay was performed using a commercially available kit (Cell Biolabs, CA, USA) by following the manufacturer's instructions. A series of diluted MDA standards was prepared, and 100 *μ*L samples were prepared after treating with PBS containing 1X butylated hydroxytoluene (BHT). Sodium dodecyl sulfate (SDS) lysis solution was added to the samples and standards, followed by 250 *μ*L of thiobarbituric acid (TBA) reagent. The samples were centrifuged at 3,000 rpm for 15 min, and the supernatant was used for analysis. MDA standards and samples were transferred to a 96-well microplate for spectrophotometric measurement at 532 nm.

#### 2.3.3. Antioxidant Enzyme Activities

Superoxide dismutase (SOD) and catalase (CAT) activities were evaluated using commercially available kits (Cayman Chemical, MI, USA) by following the manufacturer's instructions. The standards and samples were analyzed using a plate reader for spectrophotometric measurement (SOD: 450 nm, CAT: 540 nm).

#### 2.3.4. Quantification of Cyclic Guanosine Monophosphate (cGMP)

The cGMP enzyme-linked immunosorbent assay (ELISA) (Assay Designs, MI, USA) was used to determine cGMP levels. Measurements were made at 405 nm with a microplate reader.

### 2.4. Statistical Analysis

All quantitative data are reported as means ± standard deviation. Between-group comparisons were performed using the two-tailed Student *t-*test or ANOVA, followed by Tukey's test for normally distributed variables, or nonparametric analysis with a Mann–Whitney *U*-test or Kruskal-Wallis test, followed by Dunn's multiple comparison test for nonnormally distributed variables. *P* < 0.05 was considered statistically significant.

## 3. Results

### 3.1. Testicular Parameters of Spermatogenesis

Table 2 compares the histological changes in the ipsilateral and contralateral testes. Compared to the ipsilateral testes in group A, MSTDs, Johnsen scores, and the number of germ cell layers were significantly lower (*P* < 0.05) in the ipsilateral testes in groups B, F, and G than in group A and in group B compared to group C (*P* < 0.05). While ipsilateral testes in group A had normal testicular architecture and regular seminiferous tubule morphology, those in groups B, F, and G showed hypospermatogenesis, loss of germinal cells, and severely impaired seminiferous tubules. Ipsilateral testes in group C demonstrated a structure and morphology close to normal with a well-arranged cell architecture. However, no significant difference was detected in MSTD, Johnsen scores, and number of germ cell layers in the ipsilateral (groups D and E) and in the contralateral testes.

### 3.2. Detection of Peroxynitrite and Superoxide Anion

**Figure 1:**
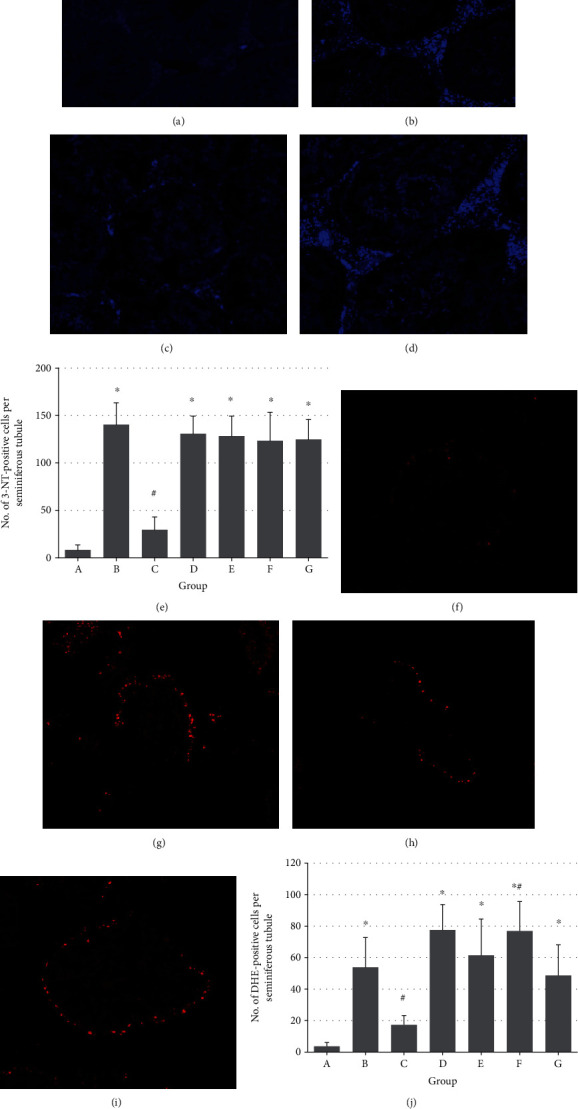
Effects of topically injected nitrite and nitrate on 3-nitrotyrosine (3-NT) and dihydroethidium (DHE) staining in the ipsilateral testis (×200). (a–d) Immunohistochemical staining of 3-NT in the left ischemic testes of groups A, B, C, and G. (e) The number of 3-NT-positive cells per seminiferous tubule in all groups. (f–i) DHE staining of in the left ischemic testes of groups A, B, C, and G. (j) The number of DHE-stained positive cells per seminiferous tubule in all groups. ^∗^*P* < 0.05 vs. group A, ^#^*P* < 0.05 vs. group B. Groups: A: control, B: ischemia/reperfusion (IR) injury, C: IR+0.12 nmol/g nitrite, D: IR+1.2 nmol/g nitrite, E: IR+12 nmol/g nitrite, F: IR+0.12 nmol/g nitrite+0.01 *μ*mol/g C-PTIO, and G: IR+0.12 nmol/g nitrate.

The number of positively stained (3-NT and DHE) cells was significantly higher in the ipsilateral testes of groups B, D, E, F, and G than in the control group (*P* < 0.05). Further, there were significantly fewer 3-NT-positive (Figures 1(a)–1(e), *P* < 0.05) and DHE-positive cells (Figures 1(f)–1(j), *P* < 0.05) in group C than in group B. Few 3-NT- and DHE-positive cells were detected in the contralateral testes (data not shown).

### 3.3. Testis Malondialdehyde (MDA) Levels

**Figure 2:**
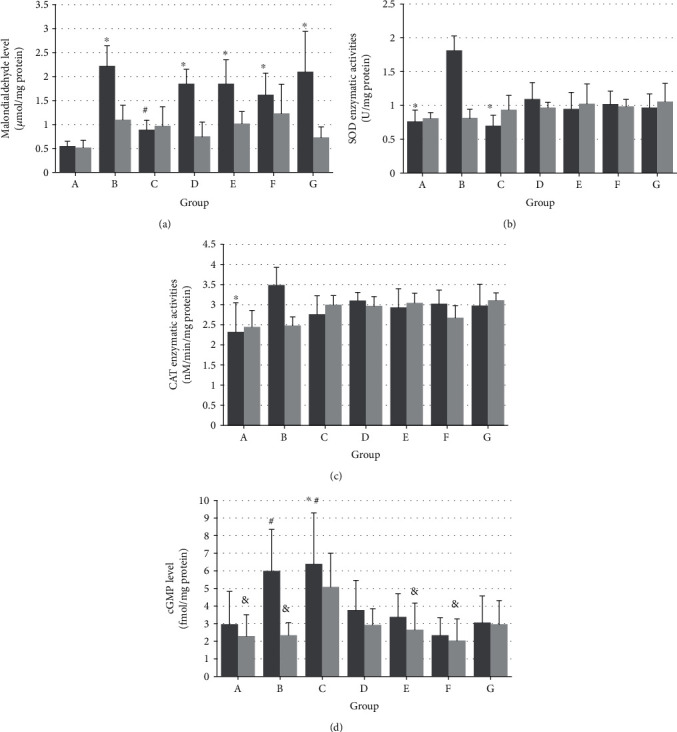
Effects of topical application of nitrite and nitrate on malondialdehyde (MDA), superoxide dismutase (SOD), catalase (CAT), and cyclic guanosine monophosphate (cGMP) levels in the ipsilateral and contralateral testes. Color black, left testis; color gray, right testis. (a) MDA values are expressed as micromoles of MDA per milligram of protein (*μ*mol/mg protein). Data are mean ± SD. ^∗^*P* < 0.05 vs. group A left testis, ^#^*P* < 0.05 vs. group B left testis. (b, c) SOD values are expressed as unit of SOD per milligram of protein (U/mg protein), and CAT values are expressed as nanomolar/minute/milligram protein. Data are mean ± SD. ^∗^*P* < 0.05 vs. group B left testis. (d) cGMP values are expressed as femtomoles of cGMP per milligram of protein (fmol/mg protein). Data are mean ± SD. ^∗^*P* < 0.05 vs. group A left testis, ^#^*P* < 0.05 vs. group F left testis, and ^&^*P* < 0.05 vs. group C right testis. Groups: A: control, B: ischemia/reperfusion (IR) injury, C: IR+0.12 nmol/g nitrite, D: IR+1.2 nmol/g nitrite, E: IR+12 nmol/g nitrite, F: IR+0.12 nmol/g nitrite+0.01 *μ*mol/g C-PTIO, and G: IR+0.12 nmol/g nitrate.

The MDA levels in the ipsilateral testes were significantly higher in groups B, D, E, F, and G than in the control group (*P* < 0.05) and in group B than in group C (*P* < 0.05) (Figure 2(a)). In contrast, there were no significant differences in the MDA levels in the contralateral testes in any of the groups.

### 3.4. Determination of Antioxidant Enzymatic Activity

SOD activity in the ipsilateral testes was significantly lower in groups A and C than in group B (*P* < 0.05) (Figure 2(b)). CAT activity in group A was significantly lower than that in group B (*P* < 0.05) (Figure 2(c)). In contrast, there were no significant differences in SOD and CAT activities in the contralateral testes in any of the groups.

### 3.5. Testicular cGMP Levels

The cGMP levels were significantly higher in the ipsilateral testes of group C than in those of groups A and F (*P* < 0.05) and in the contralateral testes of group C than in those of groups A, B, E, and F (*P* < 0.05) (Figure 2(d)).

### 3.6. Apoptosis in the Ipsilateral Testes

**Figure 3:**
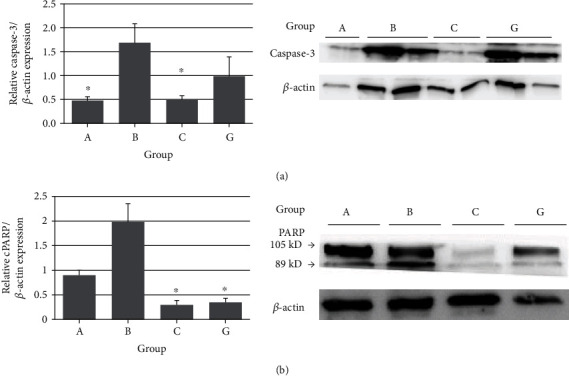
Western blot analysis of apoptosis in the ipsilateral testis: (a) the relative caspase-3/*β*-actin expression in the left testis of groups A, B, C, and G; (b) the relative cleaved PARP/*β*-actin expression in the left testis of groups A, B, C, and G. ^∗^*P* < 0.05 vs. group B. Group: A: control, B: ischemia/reperfusion (IR) injury, C: IR+0.12 nmol/g nitrite, and G: IR+0.12 nmol/g nitrate.

Caspase-3/*β*-actin levels were significantly lower in groups A and C than in group B (*P* < 0.05) (Figure 3(a)). Cleaved PARP/*β*-actin levels were significantly lower in groups C and G than in group B (*P* < 0.05) (Figure 3(b)).

## 4. Discussion

The synthesis of NO from L-arginine is catalyzed by three nitric oxide synthase isoforms via an oxygen-consuming pathway, which might be prohibited under conditions of low oxygen tension [17]. Therefore, the following alternative pathways which can reduce nitrite into nitric oxide are particularly important in ischemic conditions: deoxyhemoglobin, deoxymyoglobin, tissue heme proteins, and xanthine oxidoreductase (XOR) [18–20].

Nitrite showed protective effects in hepatic and myocardial infarction murine models [7, 21] and in cerebral I/R animal models [13]. In this study, we demonstrated that hypoxia-dependent NO production from nitrite confers cytoprotection in testicular I/R injury and that the NO scavenger, C-PTIO, suppressed the protective effect of nitrite-derived NO in ischemic testis *in vivo*. These experimental results supported our hypothesis and the results of previous studies [13, 22].

To our knowledge, this is the first study to investigate the therapeutic effects of topical application of nitrite and nitrate to testicular I/R injury. We selected the route, doses, and the time of administration based on extensive studies in liver, heart, kidney and, testis I/R injury [6, 7, 22, 23]. Because the route of administration required to achieve the best therapeutic effect was not defined and topical administration of nitrite had a therapeutic effect on rat kidneys subjected to I/R [22], we adopted a direct method, topical application, against testicular I/R injury. Although the testis is encapsulated by a fibrous envelope, tunica albuginea, this study showed that even a low dose of nitrite can penetrate this barrier.

Consistent with a previous study [24], our study demonstrated that testicular I/R induced degeneration of germ cells and impaired spermatogenesis. These effects were characterized by a significant decrease in the MSTD, number of germ cell layers, and Johnsen scores in group B compared to the control animals. It was demonstrated that ROS by ischemia-reperfusion injury has a detrimental effect on the sperm quality which contains sperm count and mobility. In addition, abnormal morphology of sperm was increased in I/R injury [25, 26]. These findings could be induced by apoptosis of testicular germ cells and overexpression of ROS. However, a low dose of topically administered nitrite (0.12 nmol/g) in this study had a remarkably protective effect on the germ cell of ipsilateral testis following I/R injury. And it could be expected that administration of nitrite could enhance sperm function in agreement with other studies [25, 26], even though we did not investigate the study regarding sperm parameters.

The administration of mid- (1.2 nmol/g) and high doses (12 nmol/g) of nitrite was ineffective while the administration of a low dose (0.12 nmol/g) of nitrite shows therapeutic effects. We thought that this adverse effect was attributed to the generation of peroxynitrite induced from the reaction of superoxide anion (O_2_-) with excessive amounts of NO in mid- and high doses of nitrite-treated groups. Because the overexpression of peroxynitrite, a strong oxidant, could result in tissue damage and apoptosis, it is concluded that NO produced in mid- and high doses of nitrite is over the threshold that can cause a harmful effect on the testis.

In this study, the activities of SOD and CAT and MDA levels in the ipsilateral testes were significantly higher in group B than in group A. These results are identical to a previous study [27]. However, there was a tendency to oppose SOD and CAT values in other ischemic tissues [28–31]. As stated in an earlier study, the testis is highly sensitive to oxidative stress [32]. Therefore, antioxidant enzymes are likely to be produced rapidly for regulating the increased level of reactive oxygen species (ROS). Fortunately, we have revealed that the application of nitrite (0.12 nmol/g) inhibited lipid peroxidation (decreasing MDA levels) and SOD and CAT activities. Similarly, the number of 3-NT- and DHE-stained cells was significantly higher in group B than in the control. Peroxynitrite and superoxide radical generation had also decreased in group C, which was consistent with previous findings [13].

Testicular I/R leads to germ cell-specific apoptosis in the rat [33]. The activation of caspase-3 leads to proteolytic cleavage of PARP, an 85 kDa fragment, which is a hallmark of apoptosis [34]. In the present study, western blotting of the germ cell lysate showed that caspase-3 and cleaved PARP levels were significantly higher in group B than in group A. However, nitrite-derived NO had an antiapoptotic effect showing the decreased level of caspase-3 and cleaved PARP in group C.

We indirectly evaluated the quantity of NO derived from nitrite by using the cGMP assay. cGMP is produced by soluble guanylyl cyclase (sGC) that is activated in the presence of NO. [35] Our results demonstrating that nitrite administration increased the cGMP levels in testicular I/R confirmed that nitrite-derived effects were mediated through a NO/sGC/cGMP pathway, which was consistent with previous findings [36]. Further investigations are needed to distinguish between NO-related compounds, such as *S*-nitrosothiols, *N*-nitrosamines, iron-nitrosyl, and nitrated lipid, which have been reported to have cytoprotective effects on I/R injury [37].

It was reported that unilateral testicular I/R adversely affects the contralateral testis, resulting in abnormal testicular structure and increasing apoptosis [38]. This has been attributed to several mechanisms including the overproduction of nitric oxide, formation of ROS, reduction of blood flow, and autoimmunization [39]. However, the validity of these hypotheses remains uncertain and is not widely accepted [40]. As our results showed that nitrite administration did not affect the contralateral testis, further studies are required to explore this contradiction.

Our study demonstrates the antioxidant and antiapoptotic effects of nitrite-derived NO under ischemic conditions and shows that the topical application of nitrite had considerable therapeutic effects on damaged testes on the basis of histopathological and biochemical results. Hence, the topical application of nitrite could be a novel and adjunctive therapeutic approach to treat testicular I/R injury.

## Figures and Tables

**Table 1:** Design of experimental study.

Group	Description	Treatment
A	Sham control	—
B	Testicular torsion (5 h)/detorsion	—
C	Testicular torsion (5 h)/detorsion	Nitrite (0.12 nmol/g, 1 min before reperfusion, t.p.)
D	Testicular torsion (5 h)/detorsion	Nitrite (1.2 nmol/g, 1 min before reperfusion, t.p.)
E	Testicular torsion (5 h)/detorsion	Nitrite (12 nmol/g, 1 min before reperfusion, t.p.)
F	Testicular torsion (5 h)/detorsion	C-PTIO (0.01 *μ*mol/g, 5 minutes before ischemia, i.v.) and nitrite (0.12 nmol/g, 1 min before reperfusion, t.p.)
G	Testicular torsion (5 h)/detorsion	Nitrate (0.12 nmol/g, 1 min before reperfusion, t.p.)

t.p.: topical application; i.v.: intravenous injection.

**Table 2:** MSTD value, Johnsen score, and number of germ cell layers in the ipsilateral and contralateral testes.

Group	MSTD		Johnsen score		Germ cell layer	
	Left	Right	Left	Right	Left	Right
A	274.10 ± 9.60	273.90 ± 6.72	8.70 ± 0.67	8.20 ± 0.79	5.40 ± 0.52	4.30 ± 0.95
B	210.70 ± 8.14^∗^	271.70 ± 8.30	2.80 ± 1.14^∗^	8.00 ± 0.82	1.40 ± 0.84^∗^	3.50 ± 0.85
C	262.10 ± 18.00^#^	271.90 ± 7.84	7.40 ± 1.17^#^	7.70 ± 0.95	5.00 ± 1.49^#^	4.20 ± 0.92
F	219.20 ± 10.97^∗^	263.90 ± 10.64	3.30 ± 1.42^∗^	7.30 ± 0.67	2.50 ± 1.08^∗^	3.90 ± 1.10
G	225.20 ± 11.98^∗^	266.40 ± 10.13	3.70 ± 1.16^∗^	7.40 ± 0.97	2.10 ± 0.74^∗^	4.40 ± 1.17

^∗^ *P* < 0.05 vs. group A, ^#^*P* < 0.05 vs. group B, MSTD: mean seminiferous tubule diameter; groups: A: control, B: ischemia/reperfusion (IR) injury, C: IR+0.12 nmol/g nitrite, F: IR+0.12 nmol/g nitrite+C-PTIO, and G: IR+0.12 nmol/g nitrate.

## Data Availability

The data used to support the findings of this study are available from the corresponding authors upon request.
